# Validity of PowerTap P1 Pedals during Laboratory-Based Cycling Time Trial Performance

**DOI:** 10.3390/sports6030092

**Published:** 2018-09-05

**Authors:** Chris Whittle, Neal Smith, Simon A. Jobson

**Affiliations:** 1 Department of Sport, Exercise and Health, University of Winchester, Sparkford Road, Winchester SO22 4NR, UK; Chris.Whittle@winchester.ac.uk; 2 Department of Sport & Exercise Sciences, University of Chichester, College Lane, Chichester PO19 6PE, UK; N.Smith@chi.ac.uk

**Keywords:** power output, cadence, power meter, mobile dynamometer

## Abstract

The use of mobile power measuring devices has become widespread within cycling, with a number of manufacturers now offering power measuring pedals. This study aimed to investigate the validity of PowerTap P1 pedals by comparing them with the previously validated Wattbike ergometer. Ten trained cyclists performed three simulated 10-mile (16-km) time trials on a Wattbike, while using PowerTap P1 pedals. There were no statistically significant differences (*p* > 0.05) between PowerTap P1 pedals and a Wattbike for maximum, minimum, and mean power output, or for maximum, minimum, and mean cadence. There were good to excellent levels of agreement between the PowerTap P1 pedals and Wattbike (ICC > 0.8) for all measured variables except minimum cadence (ICC = 0.619). This suggests that PowerTap P1 pedals provide a valid measurement of power output.

## 1. Introduction

Laboratory-based testing must be conducted upon the assumption of accurate and reliable data collection. To this end, a number of cycle ergometers have been validated for use within laboratory settings, including the Wattbike (Wattbike Ltd., Nottingham, UK), which has been shown to be both valid and reliable across a range of testing protocols.

For trained cyclist populations, the Wattbike has been reported to have a coefficient of variation (CV) of 2.6% [1] and to afford “highly reproducible” results during 30-s sprint and 4-min performance test protocols [2]. In addition, the Wattbike demonstrates high levels of intra-day and inter-day reliability [2] and no significant difference between measures of power output recorded in test–retest conditions [3]. As such, the Wattbike is considered to be an accurate and reliable tool for training and performance assessments, but there is a growing acknowledgement that laboratory-based research may not possess adequate levels of ecological validity [4,5,6,7,8,9,10,11].

Researchers have reported differences of up to 8% between indoor cycling performance and an equivalent outdoor event [8,9,10,11]. This would suggest that, despite the validity of the Wattbike, laboratory protocols do not accurately replicate ‘‘real-world’’ performance. As such, it has become increasingly important to be able to measure power output during outdoor cycling events using a range of devices designed to be fitted to the athlete’s own bicycle rather than relying only on laboratory-based measures.

The Schoberer Rad Messtechnik (SRM) device, which consists of a number of rotational strain gauges housed between the crank spindle and chain ring interface, has become the “gold standard” device for mobile power measurement applications due to its high validity and reliability [12,13,14,15] and the ability to collect valid and reliable data during actual sporting performance while using the cyclist’s own bicycle. This is not to say that it is without limitations as the SRM device remains prohibitively expensive for most recreational-level participants and there are also potential compatibility issues due to the wide range of bottom bracket standards currently employed by bicycle manufacturers. In addition, the device itself requires a certain level of mechanical competency to install correctly and requires manufacturer-based servicing for battery replacements [16]. These issues, along with the suggestion that when using this style of device there may be potential distortion of the crank arms, which would lead to systematic error in torque measurement [17], have led to the development of alternative mobile power measurement devices.

One example of this is power measuring pedals, such as Garmin Vector pedals (Garmin, Schaffhausen, Switzerland), which, instead of containing strain gauges in the crank arms, house them within each pedal body. Not only does this allow power measurement to be differentiated between right and left—something that is only possible with additional computation modules when using the SRM device—it also removes the potential influence of crank distortion. In addition, pedals-based devices are almost universally compatible, regardless of the individual bicycle componentry, which affords the potential to transfer between bicycles, with limited mechanical experience required for installation or maintenance.

Garmin Vector pedals have been compared with the SRM device and have been shown to report non-statistically significant differences in power output [16] and to give reproducible results across a range of power outputs and various cycling efforts, such as sub-maximal incremental tests, sub-maximal 30-min continuous tests and sprint tests [18]. It has been noted, however, that they increasingly overestimate at higher power outputs, whilst underestimating during sprints with a low gear ratio and during a 2-h road cycling session on hilly terrain [18]. This would suggest that data from Garmin Vector pedals should be treated with some caution.

One, largely unresearched, alternative to the Garmin Vector pedals is the P1 pedals system by PowerTap (Madison, WI, USA). The PowerTap P1 pedals have four pairs of strain gauges per pedal to measure applied force at the pedal body in both the vertical and horizontal planes and Hall effect sensors attached to the pedal axle, which results in a claimed 40 measurement points per pedal stroke [19]. In addition, the PowerTap P1 pedals have a temperature sensor at the point of force measurement. This allows for automatic accommodation for changes in temperature in an effort to avoid measurement error due to changes in environmental conditions during data collection and is something which, to the best of the authors’ knowledge, is not present in any of the other devices mentioned here.

Despite the popularity of power measuring pedals and the number of papers examining the validity of the Garmin Vector pedals, there has been little published on the validity of the PowerTap P1 pedals with, to the authors’ knowledge, only one study comparing PowerTap P1 pedals with the SRM device [20]. These researchers evaluated the pedals during both sub-maximal incremental test and sprint test protocols in a small (*n* = 5) experimental cohort. Though such protocols can provide valuable insight, it has been observed that ”constant work” or “time trial” type tests, where the cyclist is required to complete a set distance in the shortest time possible, provide more appropriate simulations of the bioenergetics of most competitive events lasting several minutes or more [21].

The aim of this study, therefore, was to assess the validity of the PowerTap P1 pedals by comparing them with the previously validated Wattbike cycle ergometer during self-paced, simulated time trials.

## 2. Materials and Methods

### 2.1. Participants

Ten trained cyclists (9 male, 1 female) (mean ± standard deviation (SD): 31 ± 10 years; 1.80 ± 0.10 m; 72 ± 9 kg, maximum power output 366 ± 69 W) volunteered to take part in the study. All cyclists held a current British Cycling Race Licence and maintained their normal diet and daily activity patterns throughout the test period. All participants gave written informed consent before taking part in the study, which had local ethics committee approval.

### 2.2. Procedure

Participants visited the laboratory on 3 separate occasions, separated by a minimum of 48 h to allow full recovery from the previous trial. Each visit consisted of a self-directed warm up followed by a simulated 10-mile (16-km) time trial and self-directed cool down. Time trials were conducted from a standing start and participants were given free choice of gearing and cadence throughout.

All trials were conducted in an air-conditioned laboratory using a standard Wattbike Pro cycle ergometer (Wattbike Ltd., Nottingham, UK), with PowerTap P1 pedals (CycleOps, Madison, WI, USA), which were zeroed before each ride, in line with manufacturer recommendations. Participants used their own cycling shoes and those who normally rode with cleats incompatible with the PowerTap pedals had their cleat position replicated with 3 bolt Kéo cleats (Look cycle international, Nevers, France). The ergometer was set to, as closely as possible, replicate the dimensions of each participant’s own bicycle.

### 2.3. Data Analysis

Power output and cadence were recorded for the duration of the time trials by a Garmin Edge 1000 head unit (Garmin, Schaffhausen, Switzerland) and the ergometer’s display unit for the PowerTap pedals and Wattbike respectively. The Garmin data were then exported to third party open source analysis software, Golden Cheetah [22], and Wattbike data was analysed using Wattbike Expert software (Wattbike Ltd., Nottingham, UK), where it was displayed as a single value per second.

Technical issues during some testing sessions meant that a small number of incomplete data sets were recorded by the Wattbike. Affected trials were removed from the study, which did not alter the number of participants tested but did result in only 20 of the 30 trials performed being analysed.

Mean, maximum, and minimum power outputs and mean, maximum, and minimum cadences were calculated, checked for normality and compared between equipment using paired samples T-tests. Effect sizes were calculated for these tests by calculating the mean difference between the two measures and then dividing the result by the pooled standard deviation.

A Bland and Altman 95% limits of agreement (LoA) analysis quantified the agreement (bias and random error) between measurement equipment. In accordance with recommendations for carrying out LoA analysis [19,23], the data were checked for heteroscedasticity via a Levene’s test and LoA analysis was followed by intra-class correlation coefficients (ICC) via the two-way mixed model to quantify the consistency of the power and cadence measurements between PowerTap P1 pedals and Wattbike.

All statistical testing was performed using IBM SPSS statistics version 24 (IMB Corporation, New York, NY, USA), with a significance level set at *p* < 0.05.

## 3. Results

Levene’s test revealed a lack of heteroscedasticity (*p* > 0.05) and the results of paired samples T-tests showed no statistically significant differences between the PowerTap P1 pedals and the Wattbike in any of the measured variables: mean power output, minimum power output, maximum power output, mean cadence, minimum cadence or maximum cadence (*p* > 0.05).

For the purpose of clarity, limits of agreement (LoA) results are reported in the format: Bias ± SD (upper confidence interval (CI), lower CI), where the bias represents the mean difference between the measurement methods and the lower and upper confidence intervals were calculated as Bias ± 1.96 × SD. This is followed by a value for the intraclass correlation coefficient (ICC).

**Figure 1:**
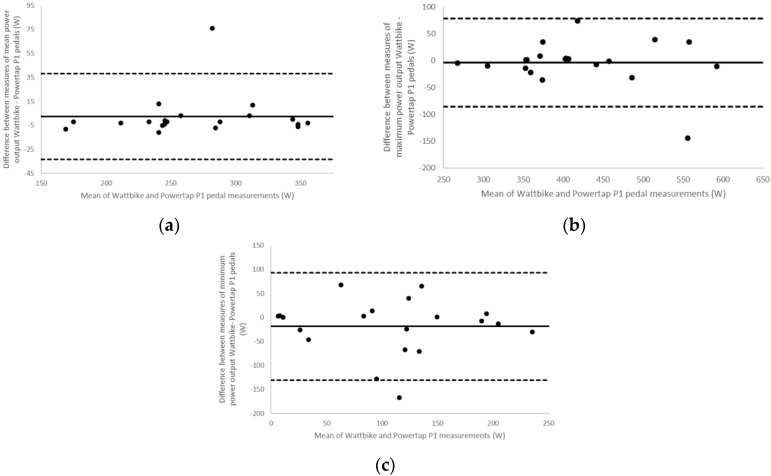
Bland-Altman plots for (**a**) mean power output (**b**) maximum power output and (**c**) minimum power output. Dashed lines represent the high and low 95% confidence intervals, the solid line shows the bias (the mean difference in power output reported between the two measurement methods).

**Figure 2:**
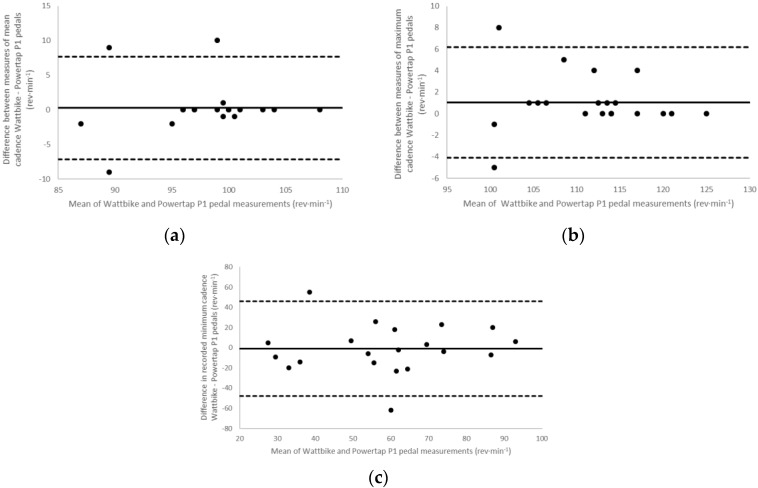
Bland-Altman plots for (**a**) mean cadence (**b**) maximum cadence and (**c**) minimum cadence. Dashed lines represent the high and low 95% confidence intervals, the solid line shows the bias (the mean difference in cadence reported between the two measurement methods).

Limits of Agreement analyses resulted in values of: 2.35 ± 18.3 W (CI: 33.5 and 38.2) and an ICC of 0.973 for mean power output (Figure 1a); −3.95 ± 41.8 W (CI: 86.0 and 78.1) and an ICC of 0.944 for maximum power output (Figure 1b) and −18.65 ± 57.2 W (CI: 130.7 and 93.4) and an ICC of 0.816 for minimum power output (Figure 1c). Cadence analysis showed 0.25 ± 3.8 rev·min^−1^ (CI: 7.2 and 7.7) and an ICC of 0.864 for mean cadence (Figure 2a); 1.05 ± 2.6 rev·min^−1^ (CI: 4.1 and 6.2) and an ICC of 0.960 for maximum cadence (Figure 2b); and −1.00 ± 23.9 rev·min^−1^ (CI: 47.8 and 45.9) and an ICC of 0.619 for minimum cadence (Figure 2c).

## 4. Discussion

The aim of this study was to assess the validity of measurements by PowerTap P1 pedals during simulated time trial performances. Difference testing suggested no statistically significant differences between the PowerTap P1 pedals and the Wattbike ergometer for any of the recorded variables.

The PowerTap P1 pedals underreported maximum power output values by 3.95 W, while overestimating mean power output values by 2.35 W in comparison to the previously validated Wattbike [1]. This represents a −0.94% difference for maximum power output and 0.88% difference for mean power output, both of which are lower than the −1.5% difference reported by Czajkowski et al. [20]. Although it is important to note that Czajkowski et al. [20] conducted both sub-maximal incremental test and sprint test protocols—in contrast to the simulated time trial used here—it would appear that there is a greater level of agreement between the Wattbike and PowerTap P1 pedals investigated in the current study than was reported between the PowerTap P1 pedals and the SRM by Czajkowski et al. [20].

In contrast, the PowerTap P1 pedals appear to have underreported minimum power output by an average of 18.65 W, a 16.03% difference between the two measurement methods. Although this appears to be a large difference, it is statistically non-significant and this variable is likely to be of little interest to cyclists in the field.

The levels of agreement shown in this study compare favourably with previously reported values [18] gathered during both submaximal incremental and continuous 30-min testing protocols to compare the data produced by Garmin Vector pedals and the SRM device. During incremental tests, non-significant differences in mean power output between devices were found [18], with LoA analysis highlighting a bias of 13.7 ± 12.4 W and 0.6 ± 6.2 W between the SRM and Stages systems and the SRM and Vector pedals, respectively. The 30-min continuous test more closely resembles the time trial effort evaluated in the current study and also produced no significant difference between the mean power outputs recorded. It was noted, however, that the Garmin Vector underestimated mean power output by 16.5% compared to the SRM. Given that a 0.88% difference for mean power output was recorded in the current study, it would appear that the PowerTap P1 pedals agree more closely with the Wattbike than do Garmin Vector pedals with the SRM.

Further support for the validity of the PowerTap P1 pedals is provided by consideration of ICC results. ICC values less than 0.5, between 0.5 and 0.75, between 0.75 and 0.9, and greater than 0.90 are suggested to be indicative of poor, moderate, good, and excellent levels of agreement between measures, respectively [24]. As such, it can be suggested that there are excellent levels of agreement between the PowerTap P1 pedals and the Wattbike for maximum cadence (0.960), maximum power output (0.944) and mean power output (0.973). These are followed by good reliability for mean cadence (0.864) and minimum power output (0.816) and moderate reliability for minimum cadence (0.619).

The differences between systems seen in this study in terms of minimum power output may be the result of a lack of synchronisation at their point of measurement as the Powertap P1 pedals claim 40 measurement points per pedal stroke [19], compared to two measurement points by the Wattbike [1]. Alternatively, the discrepancy may be the result of differences in how the two systems measure force. The Wattbike calculates force via the use of chain tension over a load cell, whereas the PowerTap P1 pedals have four pairs of strain gauges per pedal to measure applied force at the pedal body in both the vertical and horizontal planes. Regardless of the reason for this variation in measurements, these results suggest that caution should be employed when investigating minimum power output values using the PowerTap P1 pedals, although the authors would repeat that this variable is likely to be of little interest to cyclists or researchers using the PowerTap P1 pedals in the future.

It is acknowledged that the sample size for the current study (*n* = 10) could be viewed as a potential limitation. It is worth noting, however, that mean calculated effect sizes for this study were 0.11 for power output variables and 0.08 for cadence variables. With such small differences between measures it was calculated that 896 participants would be required for power output variables and 1693 for cadence variables before the level of difference seen here became statistically significant at an alpha level of *p* < 0.05.

In addition, although all participants were experienced cyclists who held a British cycling race licence, none were time trial specialists. This may have led to issues with pacing strategy and power production during the testing protocol as it has previously been shown that even competitive cyclists are not sensitive to the perceptual cues that inform their effort and ability to estimate how long it can be sustained [11]. In the current study this was not a significant concern due to the concurrent nature of the measurements. As such, the results described here would suggest that the PowerTap P1 pedals are a viable alternative to the SRM device for mobile power measurement applications.

## 5. Conclusions

There are no statistically significant differences between PowerTap P1 pedals and a Wattbike when measuring maximum, minimum, and mean power output or when measuring maximum, minimum, and mean cadence during a laboratory-based time trial. In addition, there are good to excellent levels of agreement between the PowerTap P1 pedals and Wattbike (ICC > 0.8) for all variables except minimum cadence. This study suggests that PowerTap P1 pedals are valid for measurement applications within a laboratory setting but further investigation is needed during real cycling locomotion in the field to assess their usage in outdoor applications.
